# Feeding in Tuberculosis

**Published:** 1908-03

**Authors:** James R. L. Daly

**Affiliations:** New York


### Feeding in Tuberculosis

By JAMES R. L. DALY. M. D., New York

IN tuberculosis, as in other diseases characterised by wasting of tissues, the question of feeding is recognised to be second in importance only to the relief of pain and discomfort, and then in such cases alone in which the urgency of the symptoms requires prompt action on the part of the physician for their amelioration. The very nature of the tubercular process,—that of destruction of vital structures,—supplies the indication for the employment of such measures as will assist nature in repairing, as far as possible, the waste of tissues caused by the specific microörganism. The literature on feeding in tuberculosis is voluminous enough to satisfy the most voracious medical bookworm, but there seems to be much that is useless for practical purposes, because of its ultrascientific character. In deciding as to the availability of a food in a certain diseased condition, it is of more importance to know whether it will be readily digested and be of substantial benefit to the patient, than to be able to cite how many heat units are consumed in the process of assimilation. Results should be noted in the improvement the patient derives from the ingestion of the food, and not determined merely by calorimetric estimates.

In a large percentage of cases of tuberculosis the stomach remains retentive, and the patient can partake of the same class of edibles as if in a healthy state, and without distress. It is, however, in the presence of certain disturbances that we are frequently taxed to find a suitable food which, while fulfilling the requirements of a good nutrient, will not increase the existing symptoms or retard their cure. It is then that we may have to resort to artificial nutrition to replace the more substantial but available aliments. Of the artificial food products those of a nitrogenous nature are particularly selected, because if there is to be any repair of structure damaged by disease, dependence must be placed upon the albumins. Few of the liquid preparations on the market contain a large percentage of albumins, most of them being strongly alcoholic and highly flavored beverages. Moreover, one great objection to their use lies in the sameness of taste, so that if taken for any length of time the patient rebels, and often the suggestion of the food becomes repugnant to him. In cases in which there is a tendency to diarrhea, as in tubercular ulceration of the intestines, these foods are no better digested than ordinary nourishment, and thus fail in the very purpose for which they were intended.

Milk is much frowned upon at the present time as a food for the sick, particularly in cases of intestinal disturbances. Too large an amount of it must be given to be of service for alimentation owing to the frequent nausea and vomiting to which it gives rise. While heretofore milk has been generally advised in such diseases as typhoid and tuberculosis, it is often forbidden now because if not properly digested it may act as a culture medium for the growth of bacteria. This being so, it is obvious that our old ideas of keeping a patient suffering from diarrhea on an exclusive milk diet will have to be revised. While milk contains a sufficient amount of nutriment if absorbed, there is always the possibility that absorption will not take place, that the milk will remain in the intestines undigested, and thus increase the severity of the symptoms of indigestion already present. Under these circumstances medication, aside from the use of such evacuants as act on the lower bowel, is of little avail. The course to pursue is to change the diet to some food which, while supplying the requisite amount of nourishment, will be readily absorbed by the intestines and which will not serve as a source of disturbance to the digestive functions.

In looking about for a concentrated food in asthenic cases in which diarrhea was a prominent symptom, I became interested in lacto-somatose. The point that particularly appealed to me was the slight astringency of the preparation, due to the presence of a small percentage of tannic acid in firm chemical combination with the albumoses, of which it consists. These albumoses hold an intermediary position between the albumins and peptones, and physiological investigations have shown that food proteids are largely absorbed in this form without undergoing complete peptonisation. Its astringency, I think, contributes to its more rapid absorption in diarrheal affections, and I have never found it to irritate even in cases in which milk in small amounts caused disturbances. Another valuable feature is that it can be taken in different media, according to the individual desire of the patient, and as it has practically no taste of its own, there is no such repugnance as with the variously flavored artificial foods which soon excite aversion.

After a good deal of experience with lacto-somatose it has become a favorite, and in many instances the only food which I employ in intestinal disturbances, and the following cases will serve to illustrate its efficacy in these conditions.

Case I.—E. B., aged 42 years, male, single, had never been ill before except for attacks of cold contracted at different times during the past two years. He complained of inability to partake of ordinary foods, because of diarrhea following their ingestion. He had tried various fluid preparations, but had to discontinue their use because of the troublesome sameness of diet. I placed him on a strict hygienic schedule as to exercise in breathing and walking, ventilation of sleeping-room, and so forth. For food, lacto-somatose was added to a light diet. After a period of two weeks he was able to digest heavier foods, and at present he is able to follow his occupation of driver, and shows no evidence of any intestinal disturbance.

Case II.—L. F., aged 22 years, female, single, complained of progressive loss of flesh and strength for a year, and periodic attacks of diarrhea, which later became chronic. Physical examination revealed a chronic tubercular process in scattered areas in both lungs. When I first saw the woman she was in an advanced stage of the disease, and she was kept alive for four months with lacto-somatose alone, given in a small quantity of milk, just sufficient to dissolve the preparation. After three weeks of this food in conjunction with rectal injections of tannic acid solution, the diarrhea ceased, but the patient was so reduced in vitality that she succumbed to the disease. It speaks well for the efficiency of the food that she could survive as long as she did, considering the extent of the tubercular process.

Case III.—C. K., aged 30 years, male, married, gave a history of loss of appetite; cough in morning and evening; afternoon fever, nightsweats and occasional slight attacks of hemopytsis. Physical examination showed consolidation of the upper lobe of the left lung. This young man was sent to a sanatorium close by New York City, where he developed frequent attacks of serous diarrhea. At my request the house-physician placed him on a light diet with the addition of lacto-somatose, and on every occasion when this was used the diarrhea was relieved and the patient eventually was discharged, the disease process having been completely arrested. This case was one which required for treatment simply rest in some locality away from the city, together with the proper regulation of diet. The man is of good habits, and there is every chance that he will have no further exacerbations of the disease.

CASE IV.—F. C., female, aged 42 years, married, had been suffering from attacks of intestinal indigestion for four years. She had tried most of the liquid food preparations and had been unable to continue their use because of the failure of the stomach and intestines to perform their functions, the food being passed out practically unchanged in the stools during the attack of diarrhea which would result after a few days of its use. Appropriate medication seemed to avail little until she was put on a diet of lacto-somatose. This seemed to agree with her thoroughly for she was not only enabled to retain the food and absorb it, but gained in weight and strength. She is now living in the mountains and a recent report from her attending physician states that she is not suffering from any symptoms of tuberculosis, and that the process is apparently checked, her lungs being perfectly clear of all physical signs of disease, except that of flatness over the areas previously affected, no doubt due to the formation of fibroid rings around the sites of the old lesions. This woman benefited from the use of the proper nourishment in that she was able to gain sufficient strength and vital power to be removed to a higher altitude in the country, something which was impossible earlier because of her extreme weakness.

CASE V.—C. H., laborer, aged 38 years, single, was in the advanced stage of tuberculosis, complicated with severe diarrhea, having ten to twenty stools per day before he sought treatment. His stomach was unretentive, probably due to alcoholic excesses. He was placed on a diet consisting of lacto-somatose in different media, such as beef broth, bouillon, and so forth, the fluid in just sufficient quantity to dissolve the lacto-somatose. The attacks of diarrhea ceased after ten days' treatment, and the patient, in spite of the advanced condition of the disease, did not lose ground, but rather thrived on this form of alimentation for a period of three months, after which he was removed to the home of a relative some distance from this city. Unfortunately, I was unable to follow the case or to learn anything as to the outcome.

CASE VI.—B. K., male, 38 years old, clerk, suffered from severe intestinal indigestion. After eating a serous diarrhea set in, the stools numbering ten to fifteen a day. He was unable to obtain any sort of nourishment which would be absorbed, most of the food passing through the bowel unchanged. When he came under my care I immediately placed him on a diet exclusively of lacto-somatose, and was gratified to find that this was just the variety of food necessary for one in his condition. Under the changed conditions of feeding the diarrhea gradually lessened until it was finally completely arrested. This was one of those cases in which no treatment was necessary other than proper regulation of diet. In fact, any attempt at checking the diarrhea would prove futile as long as the food partaken of was not of a nature to be readily assimilated.

These cases are but typical of the action of lacto-somatose in conditions in which diarrhea or nausea was the prominent symptom, and prevented the use of other nutriment. Its advantages in the presence of these symptoms are so marked as to make it preferable to any other method of feeding, and it should be used in every case in which intestinal disturbances are present. It is not only more readily absorbed than any other food I have employed, but its astringent effect, due to the presence of tannic acid, imparts to it a decided value in the treatment of diarrheal conditions.

850 E. One Hundred and Sixty-third Street.

